# Construction of Radial Defect Models in Rabbits to Determine the Critical Size Defects

**DOI:** 10.1371/journal.pone.0146301

**Published:** 2016-01-05

**Authors:** Ming-Dong Zhao, Jian-Shu Huang, Xin-Chao Zhang, Ke-Ke Gui, Min Xiong, Wang-Ping Yin, Feng-Lai Yuan, Guo-Ping Cai

**Affiliations:** 1 Department of Orthopaedics, Jinshan Hospital, Fudan University, Shanghai, 201508, China; 2 Department of Occupational medicine, Jinshan Hospital, Fudan University, Shanghai, 201508, China; 3 Department of Orthopaedics and Central Laboratory, the third Hospital Affiliated to Nantong University, Wuxi, Jiangsu, 214041, China; Medical University of South Carolina, UNITED STATES

## Abstract

Many studies aimed at investigating bone repair have been conducted through animal models in recent years. However, limitations do exist in these models due to varying regeneration potential among different animal species. Even using the same animal, big differences exist in the size of critical size defects (CSD) involving the same region. This study aimed to investigate the standardization of radial bone defect models in rabbits and further establish more reliable CSD data. A total of 40 6-month-old New Zealand white rabbits of clean grade totaling 80 radial bones were prepared for bone defect models, according to the principle of randomization. Five different sizes (1.0, 1.2, 1.4, 1.7 and 2.0 cm) of complete periosteal defects were introduced under anesthesia. At 12 weeks postoperatively, with the gradual increase in defect size, the grades of bone growth were significantly decreased in all 5 groups. X-ray, CT scans and H&E staining of the 1.4, 1.7, and 2.0-cm groups showed lower grades of bone growth than that of the 1.0 and 1.2-cm groups respectively (*P <* 0.05). Using rabbit radial defect model involving 6-month-old healthy New Zealand white rabbits, this study indicates that in order to be critical sized, defects must be greater than 1.4 cm.

## Introduction

Bone defects, especially segmental long-bone defects caused by nonunion, delayed union and other factors are common challenges in orthopedic treatments [1–3]. Unless the defect size was extensive, the bone defect will heal spontaneously, requiring no further intervention. However, bone tissue engineering is warranted for extensive defects that cannot heal spontaneously. Well-established experimental animal models constitute the basis for the research of bone tissue engineering [4–8].

Many studies aimed at investigating bone repair have been conducted using animal models in recent years. However, limitations exist due to varying regeneration potential among different animal species. Even though the same species is used, big differences exist in the size of a critical defect (CSD) involving the same region [5,9]. CSD is defined as the smallest size of the intraosseous wound in a particular bone and species of animal which shows less than 10% spontaneous healing during the lifetime of the animals [10–12]. The use of tissue engineering techniques to repair radial defects in New Zealand white rabbits have been reported in several recent studies [3,5,12–14]. The rabbit's forelimbs are braced by the intact ulna without internal fixation following middle radial defects. The mid-diaphyseal radius is a common site for bone defect models. However, literature reported that bone defects varying between 1.0 and 2.0 cm including sizes of 1.0 cm [15–16] to 1.2 cm [17] could not heal after a period of time. In many studies the critical size of radial defects was 1.5 cm [3,18–20]. Several researchers also reported the size of 2.0 cm [21–22] with satisfactory results. In previous studies, we found that spontaneous healing occurred when the size of radial defects (including the whole periosteum) in rabbits was between 1.0 cm and 1.2 cm. In addition to the standardization of the radial bone defect model in rabbits, more reliable CSD data for future bone repair research was also obtained.

## Materials and Methods

### Experimental animals

The animal study proposal was approved by the Institutional Animal Care and Use Committee (IACUC) of the Fudan University with the permit number: 2013-030-01. Experimental procedures were performed on all rabbits in accordance with the Regulations for the Administration of Affairs Concerning Experimental Animals approved by the State Council of People’s Republic of China. All surgery was performed under anesthesia, and all efforts were made to minimize suffering. The Experimental Animal Center of Fudan University provided 40 6-month-old, clean grade, New Zealand white adult rabbits (20 male and 20 female) with an average weight of 2.5 kg (range, 2.2–2.8 kg). Healthy rabbits were used as reported in a previous study [5]. Animals were disposed of according to the Guidance Suggestions for the Care and Use of Laboratory Animals released by the Ministry of Science and Technology of the People’s Republic of China.

A total of 80 bilateral radii were randomized. The middle segments, including the periosteum, were resected. Based on the size of defects, the middle radial defects were divided into 5 groups of 1.0, 1.2, 1.4, 1.7, and 2.0 cm.

### Establishment of the radial defect model

A total of 40 rabbits were anesthetized with diazepam (2 mg kg^-1^, SunRise Pharma, Shanghai, China) and ketamine (40 mg kg^-1^, GuTian Pharma, Fujian, China) by intramuscular injection (i.m.). Sodium Chloride (NaCl) (0.9%) (HuaLu Pharma, Shandong, China) was applied to the eyes to prevent drying. Anaesthesia was maintained by administrating 40 mg kg^-1^ ketamine by i.m. After anesthetizing, the rabbits were immobilized in a prone position. Both forelimbs were upward and shaved, disinfected with iodine and alcohol, and covered with sterile towels. An incision was made in the middle and upper section of the radial forearm, from which the skin, subcutaneous tissue and deep fascia were incised. After dissecting the muscles and exposing the radius, an orthopedic microelectric drill (Trauson Medical Instrument Co. Ltd, China) was used to remove a section of the radius including the periosteum, approximately 2.5–3.0 cm below the head of radius. Full thickness segmental defects of 1.0 cm, 1.2 cm, 1.4 cm, 1.7 cm and 2.0 cm in length, the diameter of radius in depth were created with 16 resected radii in each group. The edges of the defects were smoothed using a rasp to attain the designed size (1.0, 1.2, 1.4, 1.7, 2.0 cm) with the help of the vernier caliper. 0.5 cm sections of radioulnar interosseous membrane and 0.5 cm periosteum were removed on both sides of the broken ends. The bone fragments, related coagulation scab, and bone marrow tissues were washed with 50-ml normal saline (HuaLu Pharma, Shandong, China). After hemostasis and wound re-washing, the fascia, subcutaneous tissues and the skin incision were sutured with absorbable 4/0 surgical sutures (Ping’an Medical Equipment CO. LTD, Huai’an, China) by suturing in two layers after saline (HuaLu Pharma, Shandong, China) irrigation. The wound was disinfected with alcohol. After surgery, both forelimbs were not fixed and the rabbits were intramuscularly injected with penicillin [23–26] (130,000 U kg^-1^, HuaBei Pharma, Hebei, China) for 3 days. For analgesia, animals were dosed with subcutaneous injection of buprenorphine hydrochloride (0.03 mg kg^-1^, Drug Research Pharma, Tianjin, China) once a day for three days after the operation. The rabbits were housed separately.

### Postoperative observation

After surgery, all the animals were exposed to the natural and artificial lighting 12 h light (08:00–20:00), 12 h dark (20:00–08:00). Animals were housed at room temperature 18 ± 3°C and relative humidity (55 ± 15%). The feeding condition, weight, body temperature, breathing, appearance of surgical limbs, the possibility of incision infection and the movement function of rabbits were investigated. At 12 weeks, the animals were euthanized with an overdose of ketamine hydrochloride (GuTian Pharma, Fujian, China) and the local bone callus formation in the defect sites was observed along the original incision.

### Radiographs

Radiographs of both anterior limbs were taken in the medio-lateral projections (49 kV, 5.0 mA, 33 ms, digital X-ray machine, Siemens AG, Munich, Germany) on the first day and 12 weeks postoperatively after being anesthetized with diazepam (2 mg kg^-1^, SunRise Pharma, Shanghai, China) and ketamine (40 mg kg^-1^, GuTian Pharma, Fujian, China) by intramuscular injection (i.m.). NaCl (0.9%) (HuaLu Pharma, Shandong, China).

**Fig 1:**
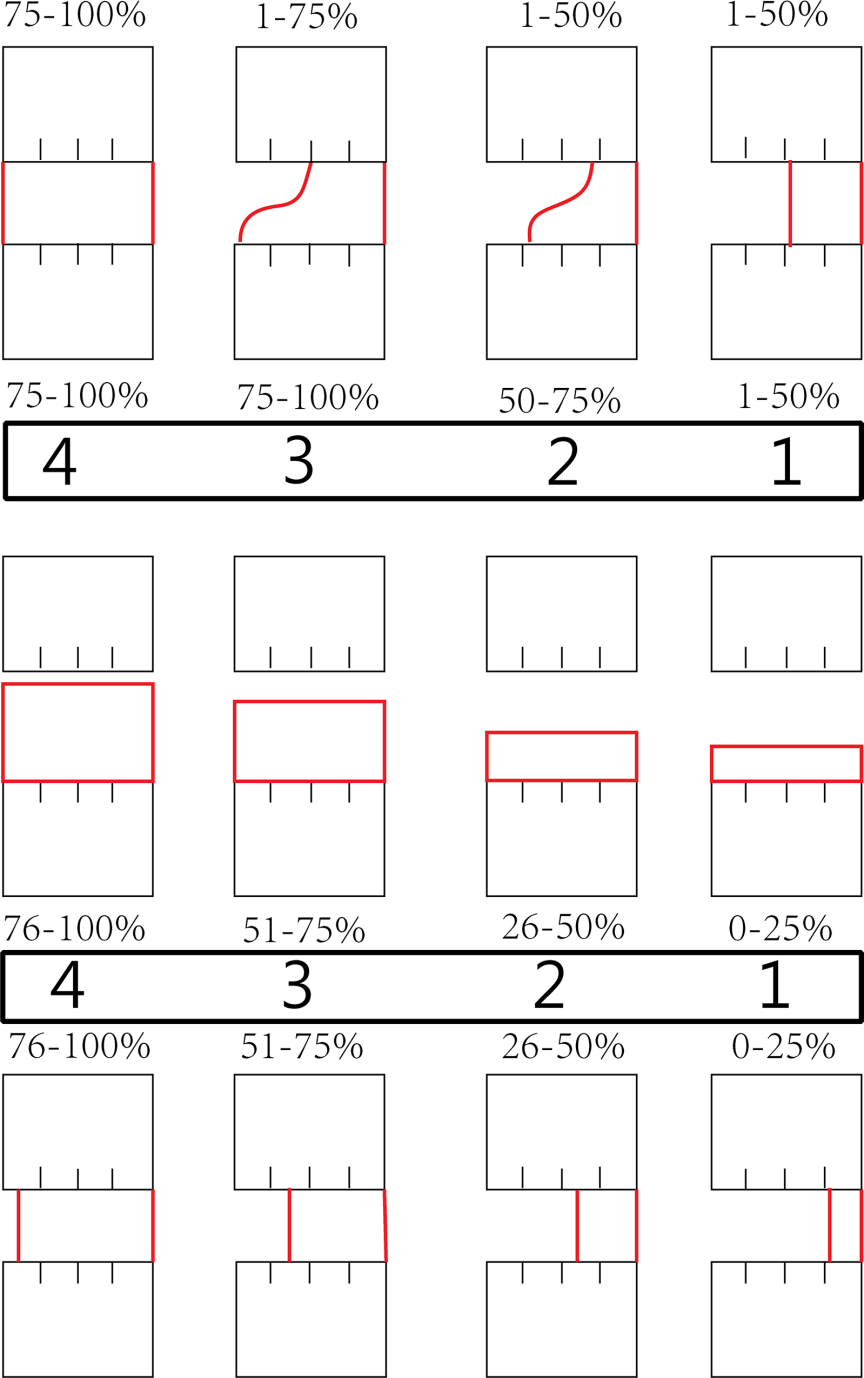
Quantitative evaluation of new bone formation in X-ray and μCT images for grades. Values refer to the percentage of the given defect border that is bridged by bone tissue of each side of the defect. A score of zero was used when no bone bridging or bone formation was found.

New bone growth was determined by evaluating the gray scale X-ray images. In Kasten’s study [3], the visually determined threshold for newly formed bone was set at 60±15. For this study a threshold was selected visually followed by the determination of a threshold for the bone and tissue of bone defects. Consequently, the ranges and means of the gray levels characteristic of the bone and soft tissue were set at 40 for all X-ray image analyses done for this study. The size of different bone defects on the first day postoperatively was measured again on the X-ray, which was compared with the former measurement during surgery to verify the exact size of different defects. According to a modified scoring system (Fig 1) [5], the connection of the broken ends in bone defects was determined using an efficacy grade to evaluate the increased density shadow in bone defects radiographically at 12 weeks. The connecting degree of new bone callus in bone defects was further determined two-dimensionally [14]. Mean grades were calculated in each group to quantify the new bone formation of the defects. The group attaining higher radiographic score was considered to have more new bone formation and be easier to spontaneous healing.

### CT scan and 3D reconstruction

Computed tomography (CT) scanning and three-dimensional (3D) reconstruction detection (80kV, 40mA, Somatom Sensation 40, Siemens AG, Munich, Germany) were performed in surgical limbs of rabbits in all groups at 12 weeks postoperatively after being euthanized with an overdose of ketamine hydrochloride (GuTian Pharma, Fujian, China), to further examine the size of the defects of radius. The threshold of new bone growth was set by the same value as that of X-ray. The efficacy grade of new bone area was evaluated using the same scoring criteria as for the radiographs [5,14].

### Histology

All animals were euthanized at 12 weeks postoperatively. A total of 80 specimens (including the adjacent ulna) from bone defect sites were fixed with 10% paraformaldehyde (XingYinHe Chemical Ltd, Hubei, China), and were decalcified by formate–sodium formate (Boster Biological Ltd, Wuhan, China). After decalcification, the samples were cut along the longitudinal plane on the microtome (BoNa Biological Ltd, Xiaogan, Hubei, China) for gross observation, then embedded in lab-grade paraffin wax (FangZheng Chemical Ltd, Sichuan, China). Four longitudinal plane sections (parallel to the long axis of bone, 5mm in thickness) from each implant was prepared and stained with hematoxylin and eosin [27].

New bone formation was quantified by the same examiner in a blind study. The region of interest (ROI) of morphometric calculations within the radius defect was based on previous studies [28–29]. The ROI was underlined with mature ulna cortical bone wall and two mature radius defect walls (proximal and distal) as borders in photomicrograph of the rabbits’ forearm longitudinal section. Mapinfo software (provided by School of Life Sciences, Fudan University, Shanghai, China) [23] was used to digitize the micrographic images. The type of tissue was identified manually, marked and assigned to a color, where bone (red) was distinguished from soft tissue (blue) using threshold setting [3,18,20,30]. The quantity of new bone formation within the ROI was determined based on gray scale in X-ray images and the threshold was set at the same value to analyze all the histological images. Four images were evaluated from each cross section. The average percentage of new bone tissue area was used to analyze the bone growth of radial defects.

### Statistical analysis

A power analysis [31–32] was done and the minimum size of sample in each group was determined to be 12. So in our study we chose 16 as the sample size of each group in our study. Statistical analyses were performed with software SPSS 13.0 (SPSS, Chicago, IL) and all data was expressed as the mean ± SEM. X-rays and CT grades of bone defects for each group were evaluated by analysis of variance (ANOVA) and Bonferroni. *P <* 0.05 was considered statistically significant.

## Results

### Number of experimental animals

Forty rabbits were included in the study. Two weeks later one rabbit died due to fracture and infection. It was replaced with another rabbit promptly. A total of 40 rabbits were analyzed.

### General conditions

**Fig 2:**
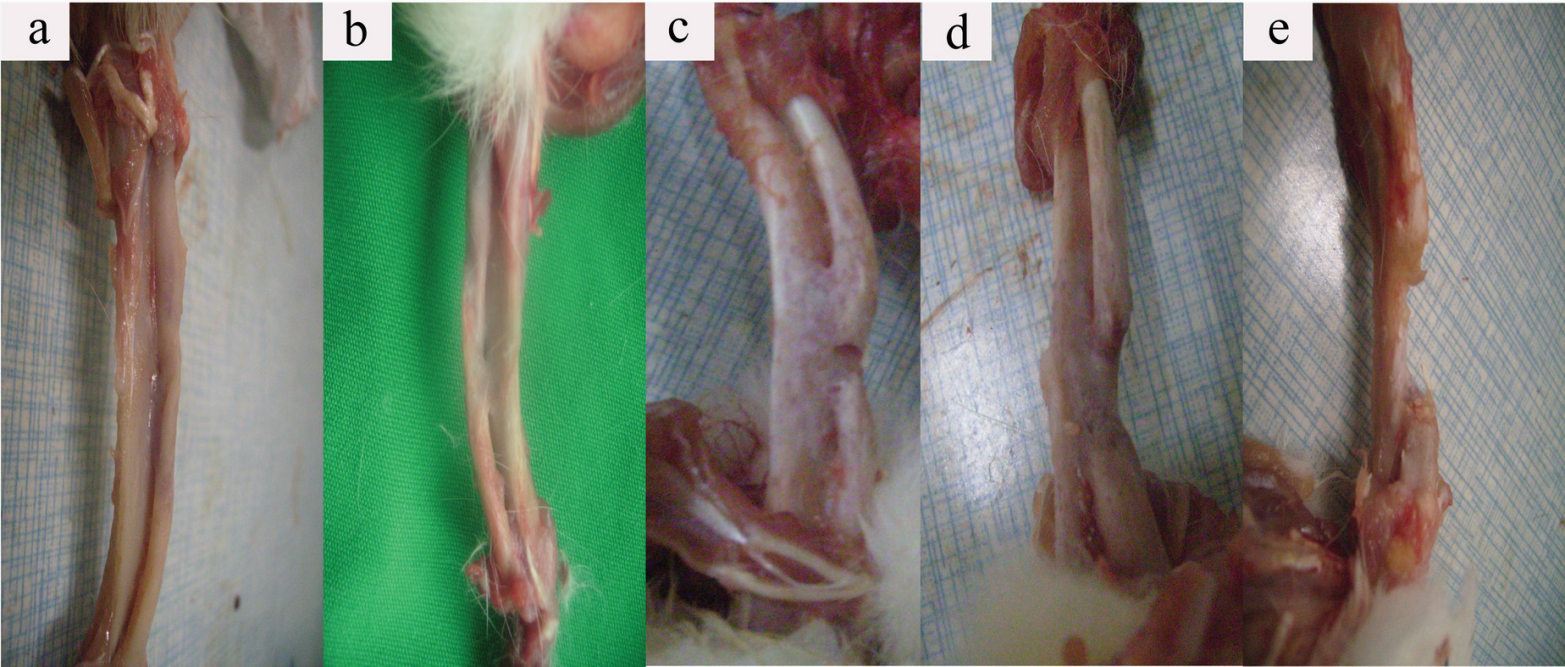
Gross observation of different radius defects after euthanasia at 12 weeks. Both the borders and center of the defects were covered by a newly formed and irregularly-shaped callus, which declined gradually with the defect size increasing (a: 1.0cm, b: 1.2cm, c: 1.4cm, d: 1.7cm, e: 2.0 cm).

All rabbits regained independent feeding ability with normal gait, following recovery from anesthesia. No apparent signs of infection such as red, hot incision or exudate were observed, other than one rabbit in the 1.2-cm group which died due to a forelimb fracture deformity and infection. At 12 weeks after surgery, in the 1.0-cm group, bone union was seen in most defective sites. Spherical calluses formed locally, with completely healed and reshaped bone accompanied by bone marrow cavity recanalization. Nonunion was only found in one sample of this group: the defective sites were filled with fibrous tissues. In the 1.2-cm group, nonunion was detected in only three samples. Significant eruption of calluses was seen in samples taken from the 1.4-cm, 1.7-cm, and 2.0-cm groups. Calluses were gradually formed from high to low with a wedge or slope shape. Occasionally, newly formed calluses were also found in the ulna that was adjacent to the defect. The most obvious defects lied in the central part where increasing calluses exist, therefore urging the calluses slope from both sides to the central of the defect. This phenomenon resulted in a bowl-shaped cross-section. The center of the defects was covered with fibrous or fat connective tissue. Both borders of the defects in the “bowl surface” were covered by newly formed and irregularly-shaped callus. The quantity of callus declined gradually with increasing defect size (Fig 2).

### Radiographs

**Fig 3:**
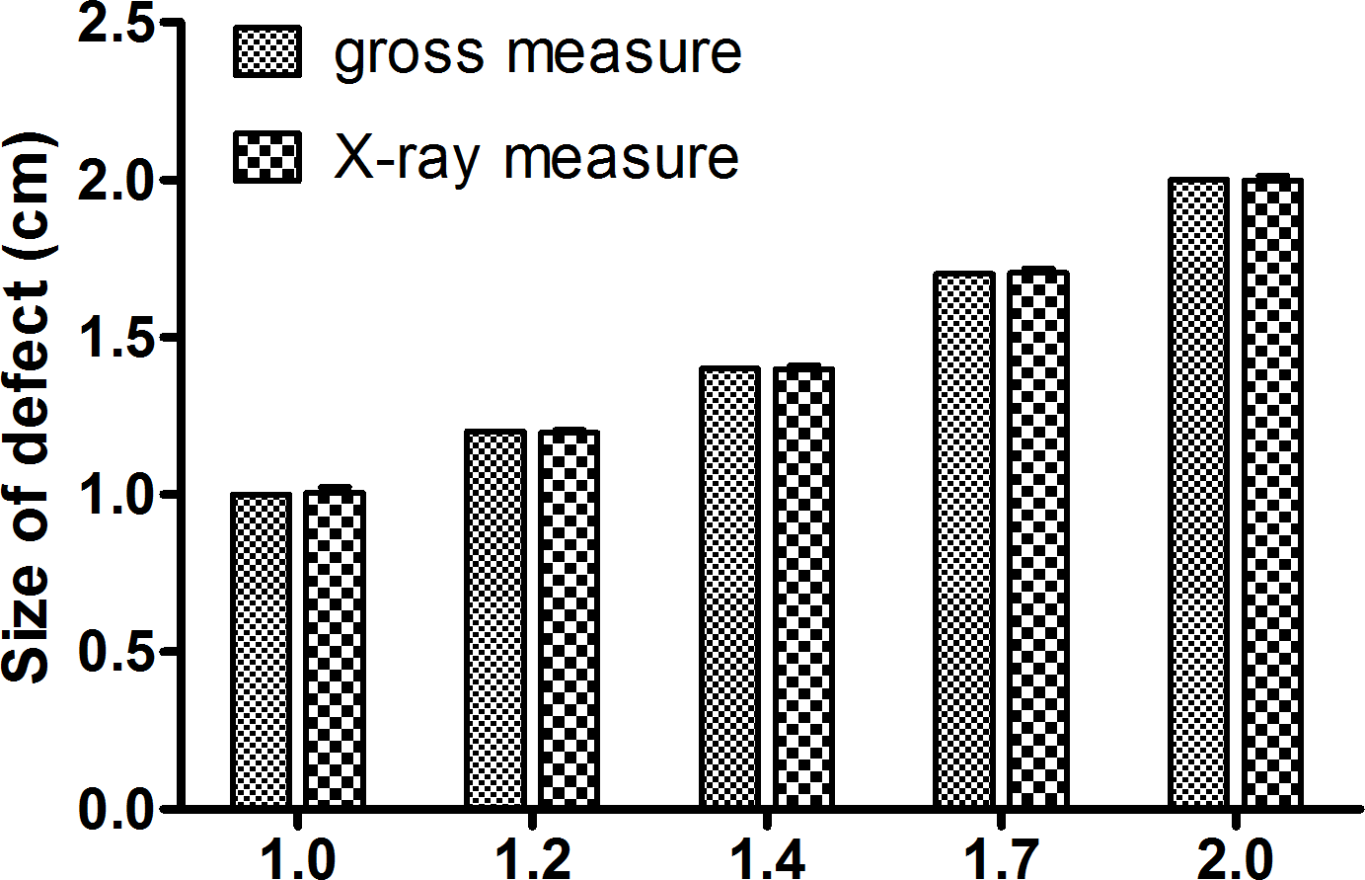
Measurement of defects on the X-ray just after operation. Size of different bone defects on the X-ray just after operation showed no statistical differences with the measurement of bone defects during operation (*P >*0.05).

**Fig 4:**
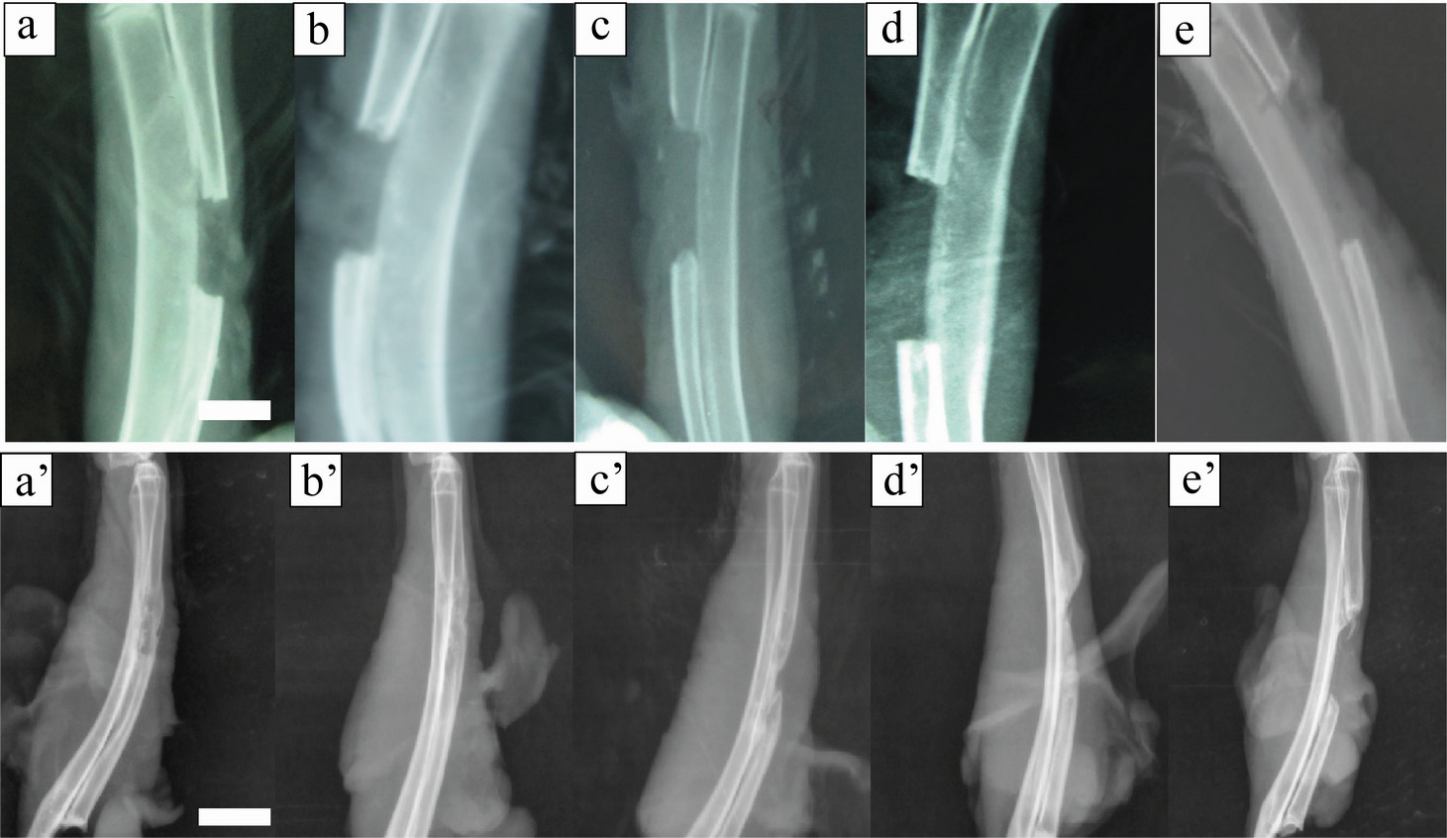
Radiograph of different radius defects just after operation and 12 weeks postoperatively. The size of the central bone defect gradually increased with respect to an increase in created defect size (a,a’: 1.0cm, b,b’: 1.2cm, c,c’: 1.4cm, d,d’: 1.7cm, e,e’: 2.0 cm, a-e: just after operation, a’-e’: 12 weeks postoperative, scale bar: 10mm).

On the first day postoperatively the size of different bone defects on the X-ray showed no statistical differences with the measurement of bone defects during operation (Fig 3, *P >*0.05). X-ray examination immediately after surgery confirmed that the size of defects in each group was 1.0–2.0 cm exactly, with no fracture involved (Fig 4A–4E).

**Fig 5:**
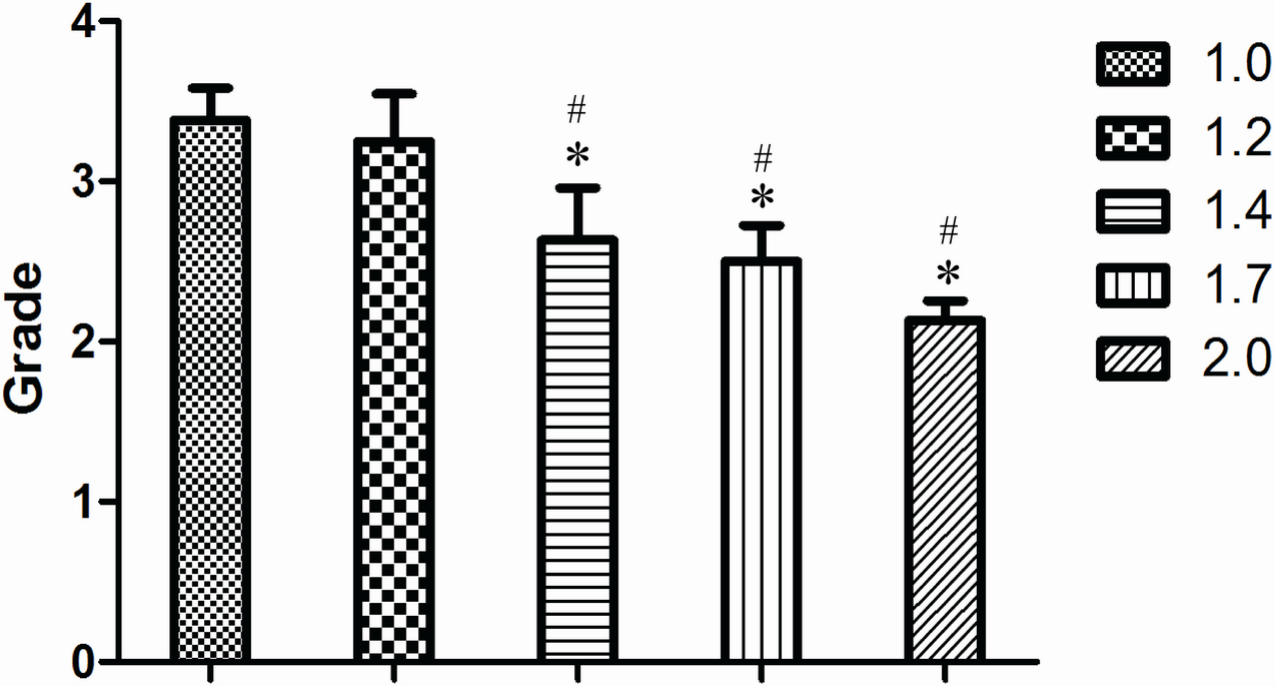
Radiographic grades of 1.0-cm and 1.2-cm groups showed more new bone formation on the X-ray. At 12 weeks postoperative, the score from X-ray images of 1.4-cm,1.7cm and 2.0-cm groups showed significantly lower new bone formation compared with the 1.0cm and 1.2cm groups (* *P <*0.05 compared with 1.0-cm groups, and # *P <*0.05 compared with 1.2-cm groups, Grade: value of score).

At 12 weeks after surgery, in most samples from the 1.0-cm and 1.2-cm groups, the defective sites were covered with callus and the ends were connected by a bony bridge. In the 1.0-cm group, most bone defects healed completely, with limited marrow recanalization. In the 1.4-cm, 1.7-cm, and 2.0-cm groups, newly-formed calluses were reduced gradually. Both the grades of new bone growth and marrow recanalization were significantly decreased with a lower bridging rate. The size of the central bone defect gradually increased with respect to an increase in created defect size (Fig 4A’–4E’). Using ANOVA, it was found that the 1.0-cm and 1.2-cm groups scored significantly higher compared with the other groups (Fig 5, *P <* 0.05).

### CT scan and 3D reconstruction

**Fig 6:**
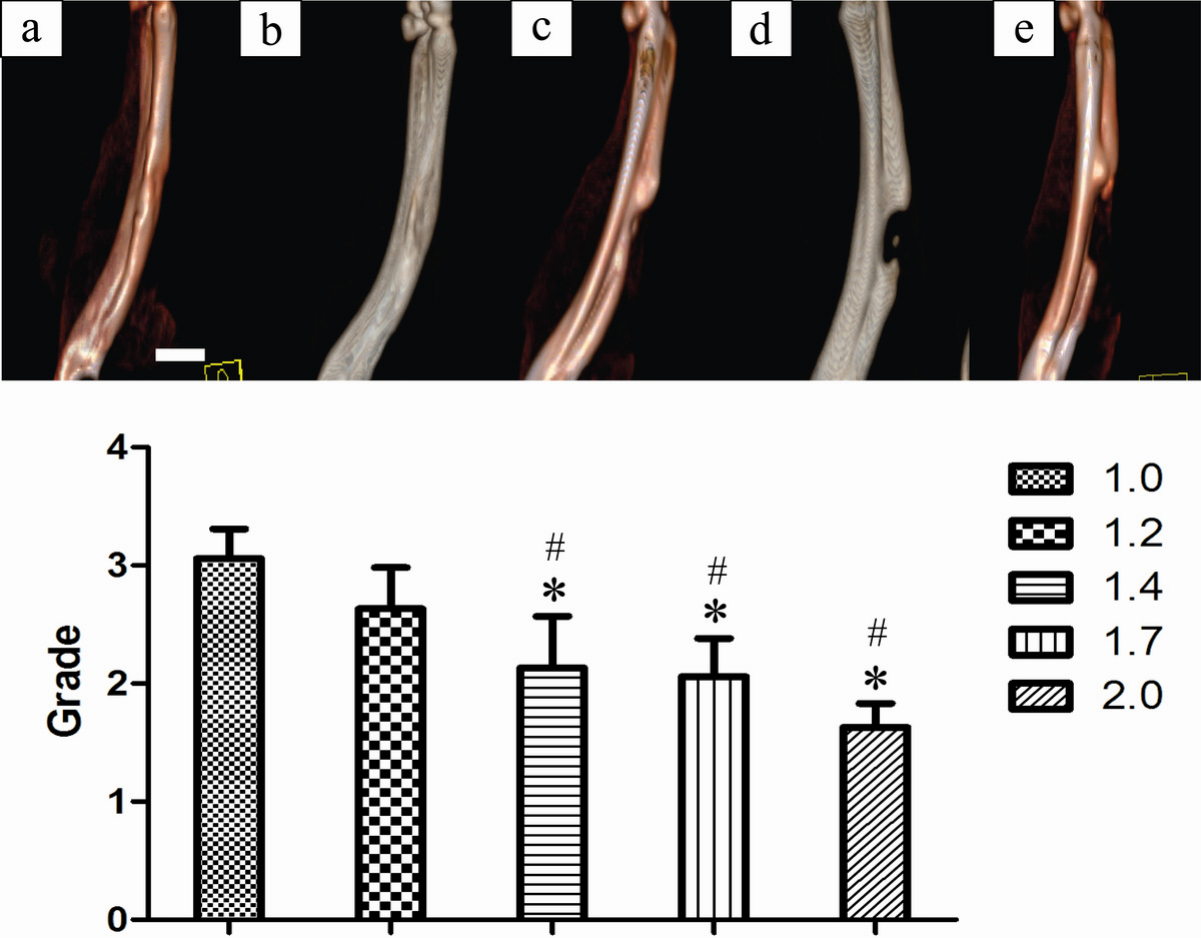
Radiographic grades of 1.0-cm and 1.2-cm groups showed more new bone formation on the Computer tomograph (CT). CT scans of different radial defects 12 weeks postoperatively. * *P <*0.05 compared with 1.0-cm groups, and # *P <*0.05 compared with 1.2-cm groups. Column graph b (1.2-cm group) showed no statistically significant difference with column graph a (1.0-cm group) (*P* >0.05). (a: 1.0cm, b: 1.2cm, c: 1.4cm, d: 1.7cm, e: 2.0 cm, scale bar: 10mm, Grade: value of score).

CT scan and 3D reconstruction were performed at 12 weeks after surgery. The defects were connected by irregular spherical callus in most samples from the 1.0-cm and 1.2-cm groups. In samples of the 1.4 cm, 1.7 cm, and 2.0 cm groups, callus connections were significantly decreased and the central defect size was specifically defined. In the 1.4 cm group, some samples showed calluses or bone or trabeculae already bridging both ends of the defect on the X-ray. However, overlapping zigzag callus and embedded soft tissue were found in defect sites by CT and 3D reconstruction, which indicated that the bone defects were still unconnected. In the 1.7-cm and 2.0-cm groups, the defect connection rate was gradually decreased and with the increase of defect size, the grades of new bone growth decreased significantly (Fig 6A–6E). Using ANOVA, the grades of 1.0-cm and 1.2-cm groups were significantly higher than that of the other groups (Fig 6, *P <*0.05).

### Histology

**Fig 7:**
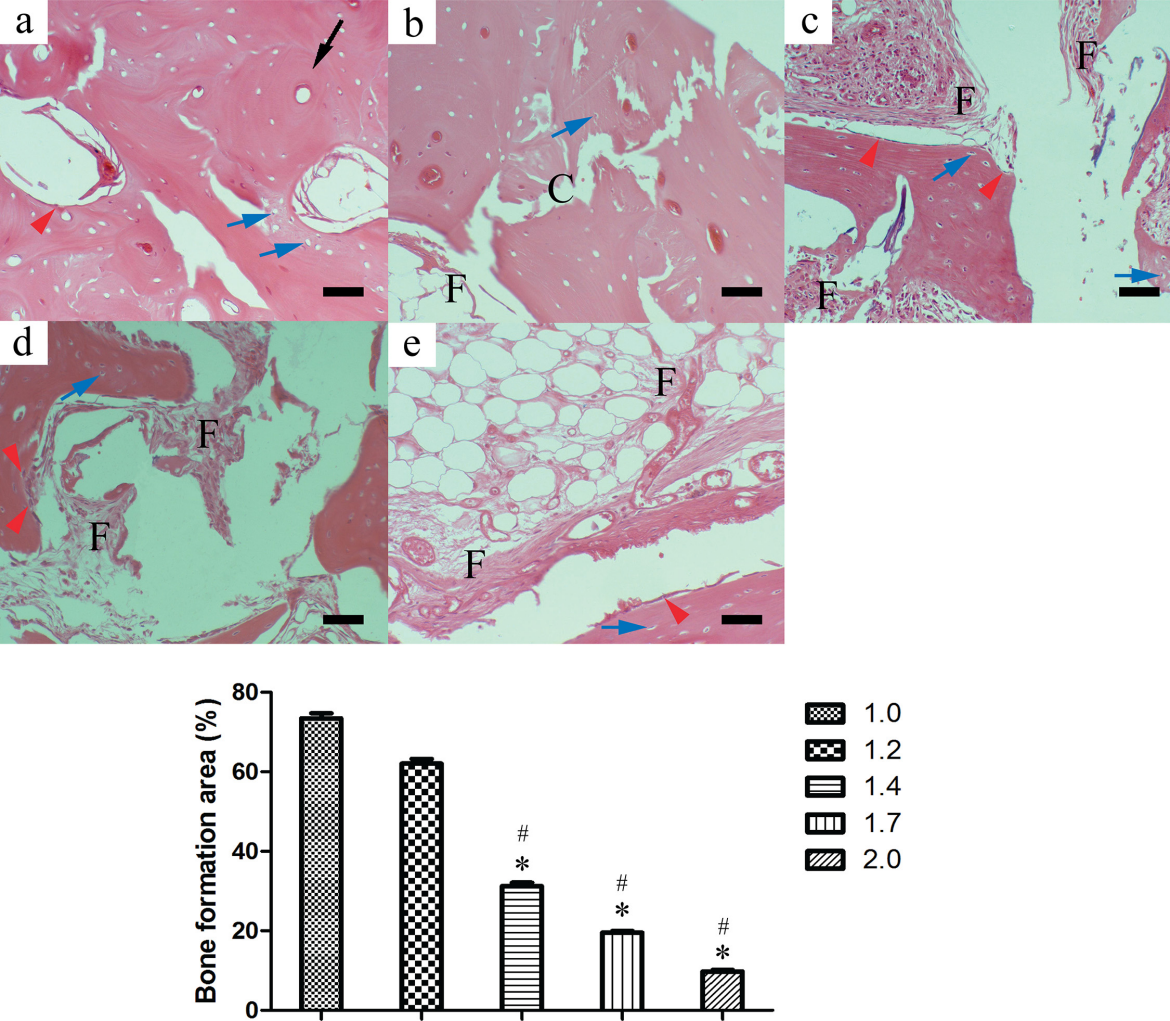
Histological assay at 12 weeks postoperative showed large amount of newly formed callus in most samples of the 1.0-cm and 1.2-cm groups. Bone tissue histology after decalcification with H&E staining at 12 weeks postoperative. The percentage of newly formed bone for the 1.0-cm and 1.2-cm groups were significantly higher than those of other groups (* *P <*0.05 compared with 1.0-cm groups, and # *P <*0.05 compared with 1.2-cm groups) (a: 1.0cm, b: 1.2cm, c: 1.4cm, d: 1.7cm, e: 2.0 cm, F: fibrous or fat tissue, C: connection of new bone formation between the two borders of bone defect, red arrow: osteoblasts, blue arrow: chondroblasts, black arrow: mature Haversian system, scale bar: 25μm).

**Fig 8:**
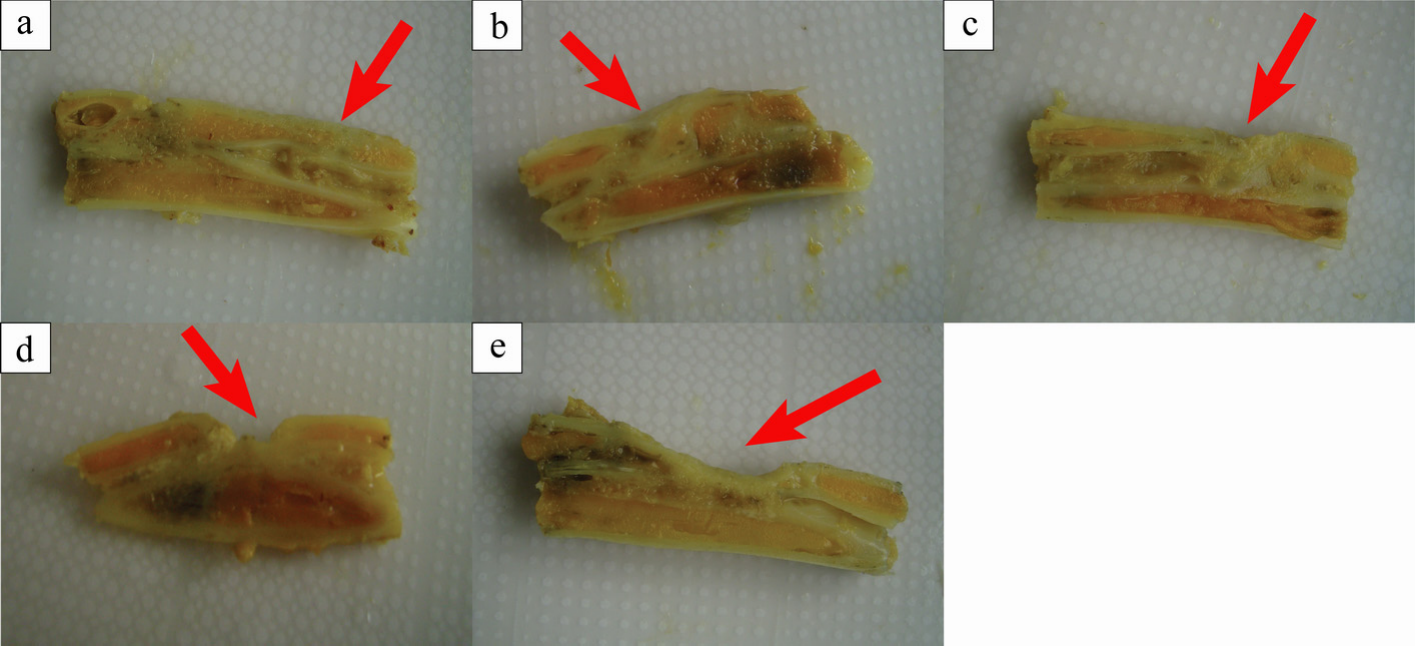
Gross observation of different radius defects cut through longitudinal section after decalcification. The central defect size was reduced with the increase of created defect size (a: 1.0cm, b: 1.2cm, c: 1.4cm, d: 1.7cm, e: 2.0 cm).

Histological assay at 12 weeks after surgery showed a large amount of newly formed callus in most samples of the 1.0-cm and 1.2-cm groups (Fig 7). Bone cells arranged regularly and in some samples the mature Haversian system was found (black arrow, Fig 7A). The arrangement of trabecular bone structure was restored. In these two groups bone defects almost completely healed, except for immature lamellar bone formation with irregular bone tissue in a few samples [7], with bone defects engulfed by fibrous or fat tissues. In tissue sections of the 1.4-cm, 1.7-cm, and 2.0-cm groups, medullary cavities were enclosed by immature newly formed woven bones or bone-like tissues. Trabecular bone formation was decreased toward the defective center. Periosteal reaction and thick new bone-like tissues were found at the bottom of the defects. Osteoblasts and chondroblasts could be detected in all five groups (red and blue arrow, Fig 7), which showed this area should be immature bone tissue, i.e., this part should be the new bone tissue [33–34]. The central defect size was reduced with the increase of created defect size (Fig 8). The area percentage of newly formed bone in 1.0-cm and 1.2-cm groups showed significantly higher than those in other groups (Fig 7, *P <*0.05).

## Discussion

According to ASTM Standard F2721, CSD models in animal experiments for the tissue engineered constructs are defined as “a defect that will not heal without intervention”. They have been described for small (i.e., mouse, rat, rabbit) and large (i.e., dog, sheep, pig) animals. Generally large animal models are advantageous in terms of the dimensions and biomechanical situations, however, they are labor, time, and cost intensive. Small animal models could avoid the demerits of large animals, especially for large sample sizes, so they are considered to be more suitable for fundamental research problems and screening experiments [35]. For example, CSD in the femur of Wistar rats is 5 mm [35], in the femur of C57BL/7 mice is 2.5mm [36], while in an adult guinea pig, the CSD (diameter) within the calvaria is 8mm [37], and in dogs the CSD of the ulnar is 2.0 cm [38]. In recent years, the CSD of radial bone defects in New Zealand white rabbits were largely applied to tissue engineering research. In this study, we selected an identical number of male and female rabbits aged six months, with similar weights and who were raised in the same environment. At 12 weeks post CSD surgery, we conducted radiological and 3D CT examination as well as observing the clinical signs, such as body temperature, breathing, movement, weight, food and water consumption, assessment of the incision, gross observation of the defect site and tissue sections. It was found that a high chance of spontaneous healing occurred when the defect size was 1.0 cm or 1.2 cm. When the defect size was above 1.4 cm, the grades of bone growth were significantly less compared with the 1.0-cm and 1.2-cm groups. This indicated 1.4 cm was more consistent with the requirement of tissue engineering models.

Defect size is one determinant of the degree of bone healing. The experimental animals’ age, weight, and gender, also affected the rate of union [39]. Bolander indicated that the middle of the full periosteum should be used as the defect site in the radial defect model and the size was at least twice the diameter [40]. Johnson showed that complete bone defects need to occur under an environment that was not conducive to bone growth. For example, if the defect size was more than 3–4 times the diameter of the backbone, or associated with less red bone marrow and partly covered with less muscle, spontaneous healing does not occur [41]. The average diameter of radii measured in this study was 0.2 cm-0.4 cm. Zhang selected 1.0 cm as the CSD of middle radial defects in rabbits [15] and Xu selected 1.2 cm [17]. In both cases, no spontaneous healing was detected. Most experts preferred 1.5 cm as the size of middle radial defect. Kasten [3], Niemeyer [18], and Geiger [19] created a radial defect model by resecting a 1.5-cm segment in the distal radius (including the periosteum). They removed periosteum 0.5 cm both proximal and distal to the defect. At 16 weeks after surgery, no bone union was seen and the broken ends were enclosed with a small amount of newly formed callus, which was in line with the standard of bone defect models. Cheng [25] made a 1.5-cm radial segment defect (including the periosteum) after which complete hemostasis was performed. At 12 weeks after surgery, no bone connection was found and the defect sites were filled with fibrous scar tissues. In Cheng’s research [25], 800,000 unit of penicillin was administrated each day for 3 continuous days postoperatively. Penicillin has also been used in other studies [23–24,26]. The animals in their studies survived during the study period with no evidence of inflammation or infection at the implantation site. However, whether antibiotic had influence to bone growth in the defects is under discussion. To address this question, some scholars have done related research. Sculean applied a randomized, controlled, blinded, clinical investigation to determine the effect of postsurgical antibiotics on the healing of intrabony defects following treatment with enamel matrix proteins [42]. The test group received a combination of systemic antibiotics consisting of amoxicillin and metronidazole daily for 7 days starting the day of surgery, whereas the control group did not receive any antibiotics. No statistically significant differences between the 2 treatment modalities in any of the investigated clinical parameters were found at 1 year after therapy. They concluded that the treatment of intrabony defects with enamel matrix proteins with or without postoperative administration of antibiotics resulted in the same good clinical outcome, which also suggested amoxicillin and metronidazole had little to do with the healing of bone defect. All the factors affecting new bone growth should be avoided as much as possible while studying CSD. In view of routine clinical therapy for infection and previous studies, we used penicillin 130,000 U kg-1 for each rabbit postoperatively for 3 days in our animal experiment. Under this treatment, one animal had to be removed from the study, but there were no notable inflammation or infection indicators in any of the other animals. On the other hand, age should be an important factor in bone regenerative behavior for experimental animals [5]. However, some previous studies did not mention the animal age when dealing with critical-sized radial defects [43–44], or only focused on radiographs that the epiphyseal plates were closed [22,45]. To certify the importance of age in animal experiments, Bodde conducted research on 1.5-cm and 2.0-cm radial defect models in 4-month-old New Zealand white rabbits [5]. At 12 weeks after surgery in their study, analysis of radiographs, 3D CT reconstructions, and histology indicated that 6 out of 16 cases of 2.0-cm bone defect and 7 out of 15 cases of 1.5-cm bone defects showed a state of nonunion. Typical New Zealand white rabbits mature and experience complete skeletal growth between 19 and 32 weeks [46–47]. However, four-month old New Zealand white rabbits have been found to reach approximately 95% of their adult length [48]. For this study, the high incidence of nonunion suggests that the subject rabbits were not mature and still possessed a high potential of bone regeneration similar to newborn animals [5,49]. Therefore, it was concluded that healing of bone defects was easier in younger rabbits. It was suggested that the rabbits selected for experiments should be at least 6- months old. While bone remodeling in humans takes approximately 2 to 8 months [50], this timeframe can be much shorter in animals [49]. Therefore, in our present study the observation period of bone defects was 12 weeks, in accordance with previous research [5].

In previous studies, CSD changed greatly from 1.0 cm to 2.0 cm. It was found that the bone growth in 1.0-cm and 1.2-cm rabbit radial defects (the full periosteum) was greater. Based on previous studies in which such sizes were applied, we further investigated the critical size of middle radial bone defect in rabbits. We used 40 6-month-old New Zealand white adult rabbits (20 male and 20 female) with an average weight of 2.5 kg. Rabbits were fed by the same breeder and surgeries were performed on the same day. We created radial defects (including the full periosteum) of 1.0 cm, 1.2 cm, 1.4 cm, 1.7 cm, and 2.0 cm [51–52]. In addition, the interosseous membranes were removed to minimize the possible deviation or margin of error in spontaneous healing caused by the presence of periosteum and interosseous membranes. At 12 weeks after surgery, results of X-ray, CT and pathological scoring indicated that in the 1.0-cm and 1.2-cm groups, the grades of new bone growth in the defects were higher than that of other three groups. In some samples, bone marrow recanalization was detected, differing from previous reports. In groups with a defect size above 1.4 cm, the grades of bone growth in the defects were significantly decreased. In the 2.0-cm group, there was only one case in which the callus seemed to be connected, but bone nonunion was still detected with CT and pathological sections. The common radiographic criteria was recently adopted in the clinical trials of fracture-healing, meanwhile the bias of radiographic score would be unavoidable. The healing of the defects in the rabbits’ radii by injection of the fluorochrome labeling such as calcein twice before necropsy has been utilized in the recent studies [53]. The process of bone neodeposition could be revealed by intravital marker with fluorescence microscopy, as well as the course of bone defect healing process in the examined subjects [11]. To calculate the mineralized surface per bone surface, researchers have applied mineral apposition rate and bone formation rates relative to bone surface or total volume, respectively [54]. However, the new bone growth in bone defects could approximately be evaluated by our radiographic scoring system and static histomorphometry. Future studies aiming at dynamic histomorphometry using calcein green injections would make measurement of new bone formation more accurate.

The most important and novel findings in the present study are as follows: (1) In defect sizes less than 1.2 cm, the grade of bone growth was relatively high; (2) The grade of bone growth in groups with a defect greater than 1.4-cm was much lower than that of the 1.0 and 1.2 cm groups respectively (*P <* 0.05). Therefore, in bone tissue engineering, models with radius defects greater than 1.4 cm (involving periosteum) have been proved to be the critical size.

## References

[1] Schmidmaier G, Capanna R, Wildemann B, Beque T, Lowenberg D (2009) Bone morphogenetic proteins in critical-size bone defects: what are the options? Injury 40, Supplement 3: S39–S43. 20082789 10.1016/S0020-1383(09)70010-5

[2] Amorosa LF, Lee CH, Aydemir AB, Nizami S, Hsu A, Patel NR, et al (2013) Physiologic load-bearing characteristics of autografts, allografts, and polymer-based scaffolds in a critical sized segmental defect of long bone: an experimental study. Int J Nanomedicine 8: 1637–1643. 10.2147/IJN.S42855 23637532 PMC3639117

[3] Kasten P, Vogel J, Geiger F, Niemeyer P, Luginbuhl R, Szalay K (2008) The effect of platelet-rich plasma on healing in critical-size long-bone defects. Biomaterials 29: 3983–3992. 10.1016/j.biomaterials.2008.06.014 18614227

[4] Schmitz JP, Hollinger JO (1986) The critical size defect as an experimental model for craniomandibulofacial nonunions. Clin Orthop Relat Res: 299–308. 3084153

[5] Bodde EW, Spauwen PH, Mikos AG, Jansen JA (2008) Closing capacity of segmental radius defects in rabbits. J Biomed Mater Res A 85: 206–217. 17688264 10.1002/jbm.a.31549

[6] Caporali EH, Rahal SC, Morceli J, Taga R, Granjeiro JM, Cestari TM, et al (2006) [Assessment of bovine biomaterials containing bone morphogenetic proteins bound to absorbable hydroxyapatite in rabbit segmental bone defects]. Acta Cir Bras 21: 366–373. 17160247 10.1590/s0102-86502006000600003

[7] Kokubo S, Fujimoto R, Yokota S, Fukushima S, Nozaki K, Takahashi K, et al (2003) Bone regeneration by recombinant human bone morphogenetic protein-2 and a novel biodegradable carrier in a rabbit ulnar defect model. Biomaterials 24: 1643–1651. 12559824 10.1016/s0142-9612(02)00551-3

[8] El-Ghannam A, Cunningham LJ, Pienkowski D, Hart A (2007) Bone engineering of the rabbit ulna. J Oral Maxillofac Surg 65: 1495–1502. 17656274 10.1016/j.joms.2006.10.031

[9] Reichert JC, Saifzadeh S, Wullschleger ME, Epari DR, Schutz MA, Duda GN, et al (2009) The challenge of establishing preclinical models for segmental bone defect research. Biomaterials 30: 2149–2163. 10.1016/j.biomaterials.2008.12.050 19211141

[10] Gugala Z, Gogolewski S (1999) Regeneration of segmental diaphyseal defects in sheep tibiae using resorbable polymeric membranes: a preliminary study. J Orthop Trauma 13: 187–195. 10206250 10.1097/00005131-199903000-00006

[11] Cacchioli A, Spaggiari B, Ravanetti F, Martini FM, Borghetti P, Gabbi C (2006) The critical sized bone defect: morphological study of bone healing. Ann Fac Med Vet di Parma 26: 97–110.

[12] Zhao M, Zhou J, Li X, Fang T, Dai W, Yin W, et al (2011) Repair of bone defect with vascularized tissue engineered bone graft seeded with mesenchymal stem cells in rabbits. Microsurgery 31: 130–137. 10.1002/micr.20854 21268110

[13] Pekkarinen T, Jamsa T, Maatta M, Hietala O, Jalovaara P (2006) Reindeer BMP extract in the healing of critical-size bone defects in the radius of the rabbit. Acta Orthop 77: 952–959. 17260207 10.1080/17453670610013286

[14] Hedberg EL, Kroese-Deutman HC, Shih CK, Lemoine JJ, Liebschner MA, Miller MJ, et al (2005) Methods: a comparative analysis of radiography, microcomputed tomography, and histology for bone tissue engineering. Tissue Eng 11: 1356–1367. 16259591 10.1089/ten.2005.11.1356

[15] Zhang Y, Lu S, Wang J, Zhang B (1999) [Expression of BMP-2, TGF-beta, bFGF in guided bone regeneration]. Zhonghua Wai Ke Za Zhi 37: 399–402. 11829871

[16] Gogolewski S, Pineda L, Busing CM (2000) Bone regeneration in segmental defects with resorbable polymeric membranes: IV. Does the polymer chemical composition affect the healing process? Biomaterials 21: 2513–2520. 11071601 10.1016/s0142-9612(00)00119-8

[17] Xu YT, Gu JF, Shang P (2001) [Experimental study of bone repair induced by cryopreserved allograft periosteum and fetal bone composition in bone defect]. Zhongguo Xiu Fu Chong Jian Wai Ke Za Zhi 15: 183–187. 11393964

[18] Niemeyer P, Szalay K, Luginbuhl R, Sudkamp NP, Kasten P (2010) Transplantation of human mesenchymal stem cells in a non-autogenous setting for bone regeneration in a rabbit critical-size defect model. 900–908 p. 10.1016/j.actbio.2009.09.007 19766744

[19] Geiger F, Lorenz H, Xu W, Szalay K, Kasten P, Claes L, et al (2007) VEGF producing bone marrow stromal cells (BMSC) enhance vascularization and resorption of a natural coral bone substitute. Bone 41: 516–522. 17693148 10.1016/j.bone.2007.06.018

[20] Beck A, Woods S, Lansdowne JL, Arens D (2013) The effects of multiple high-resolution peripheral quantitative computed tomography scans on bone healing in a rabbit radial bone defect model. Bone 56: 312–319. 10.1016/j.bone.2013.06.025 23827347

[21] Seeherman HJ, Azari K, Bidic S, Rogers L, Li XJ, Hollinger JO, et al (2006) rhBMP-2 delivered in a calcium phosphate cement accelerates bridging of critical-sized defects in rabbit radii. J Bone Joint Surg Am 88: 1553–1565. 16818982 10.2106/JBJS.E.01006

[22] Zegzula HD, Buck DC, Brekke J, Wozney JM, Hollinger JO (1997) Bone formation with use of rhBMP-2 (recombinant human bone morphogenetic protein-2). J Bone Joint Surg Am 79: 1778–1790. 9409791 10.2106/00004623-199712000-00003

[23] Zhou J, Lin H, Fang T, Li X, Dai W, Uemura T, et al (2010) The repair of large segmental bone defects in the rabbit with vascularized tissue engineered bone. Biomaterials 31: 1171–1179. 10.1016/j.biomaterials.2009.10.043 19880177

[24] Shimizu T, Akahane M, Morita Y, Omokawa S, Nakano K, Kira T, et al (2015) The regeneration and augmentation of bone with injectable osteogenic cell sheet in a rat critical fracture healing model. Injury. 10.1016/j.injury.2015.04.031 26021664

[25] Cheng L, Lu X, Shi Y, Li L, Xue J, Zhang L, et al (2012) Repair of segmental bone defects with bone marrow and BMP-2 adenovirus in the rabbit radius. Applied Surface Science 262: 188–193.

[26] Bronoosh P, Tanideh N, Noorafshan A, Andisheh TA, Aalipanah M, Kamali F, et al (2015) Effects of low-intensity pulsed ultrasound on healing of mandibular bone defects: an experimental study in rabbits. Int J Oral Maxillofac Surg 44: 277–284. 10.1016/j.ijom.2014.09.020 25448406

[27] Cao L, Wang J, Hou J, Xing W, Liu C (2014) Vascularization and bone regeneration in a critical sized defect using 2-N,6-O-sulfated chitosan nanoparticles incorporating BMP-2. Biomaterials 35: 684–698. 10.1016/j.biomaterials.2013.10.005 24140042

[28] Thor AL, Hong J, Kjeller G, Sennerby L, Rasmusson L (2013) Correlation of platelet growth factor release in jawbone defect repair—a study in the dog mandible. Clin Implant Dent Relat Res 15: 759–768. 10.1111/j.1708-8208.2011.00405.x 22235990

[29] Batista JD, Sargenti-Neto S, Dechichi P, Rocha FS, Pagnoncelli RM (2015) Low-level laser therapy on bone repair: is there any effect outside the irradiated field? Lasers Med Sci. 10.1007/s10103-015-1752-3 25975746

[30] Sohn JY, Park JC, Um YJ, Jung UW, Kim CS, Cho KS, et al (2010) Spontaneous healing capacity of rabbit cranial defects of various sizes. J Periodontal Implant Sci 40: 180–187. 10.5051/jpis.2010.40.4.180 20827327 PMC2931306

[31] Chow SC, Wang H (2001) On sample size calculation in bioequivalence trials. J Pharmacokinet Pharmacodyn 28: 155–169. 11381568 10.1023/a:1011503032353

[32] Kieser M, Hauschke D (1999) Approximate sample sizes for testing hypotheses about the ratio and difference of two means. J Biopharm Stat 9: 641–650. 10576408 10.1081/bip-100101200

[33] Xu F, Ding H, Song F, Wang J (2014) Effects of preparation methods on the bone formation potential of apatite-coated chitosan microspheres. J Biomater Sci Polym Ed 25: 2080–2093. 10.1080/09205063.2014.970604 25324120

[34] Kim HC, Song JM, Kim CJ, Yoon SY, Kim IR, Park BS, et al (2015) Combined effect of bisphosphonate and recombinant human bone morphogenetic protein 2 on bone healing of rat calvarial defects. Maxillofac Plast Reconstr Surg 37: 16 26161381 10.1186/s40902-015-0015-3 PMC4488498

[35] Poser L, Matthys R, Schawalder P, Pearce S, Alini M, Zeiter S (2014) A standardized critical size defect model in normal and osteoporotic rats to evaluate bone tissue engineered constructs. Biomed Res Int 2014: 348635 10.1155/2014/348635 24738053 PMC3967594

[36] Cheng BH, Chu TM, Chang C, Kang HY, Huang KE (2013) Testosterone delivered with a scaffold is as effective as bone morphologic protein-2 in promoting the repair of critical-size segmental defect of femoral bone in mice. PLoS One 8: e70234 10.1371/journal.pone.0070234 23940550 PMC3733987

[37] Gosain AK, Song L, Yu P, Mehrara BJ, Maeda CY, Gold LI, et al (2000) Osteogenesis in cranial defects: reassessment of the concept of critical size and the expression of TGF-beta isoforms. Plast Reconstr Surg 106: 360–371, 372. 10946935 10.1097/00006534-200008000-00018

[38] Minier K, Toure A, Fusellier M, Fellah B, Bouvy B, Weiss P, et al (2014) BMP-2 delivered from a self-crosslinkable CaP/hydrogel construct promotes bone regeneration in a critical-size segmental defect model of non-union in dogs. Vet Comp Orthop Traumatol 27: 411–421. 10.3415/VCOT-14-03-0036 25327869

[39] Aalami OO, Nacamuli RP, Lenton KA, Cowan CM, Fang TD, Fong KD, et al (2004) Applications of a mouse model of calvarial healing: differences in regenerative abilities of juveniles and adults. Plast Reconstr Surg 114: 713–720. 15318051 10.1097/01.prs.0000131016.12754.30

[40] Bolander ME, Balian G (1986) The use of demineralized bone matrix in the repair of segmental defects. Augmentation with extracted matrix proteins and a comparison with autologous grafts. J Bone Joint Surg Am 68: 1264–1274. 3533947

[41] Johnson EE, Urist MR, Schmalzried TP, Chotivichit A, Huang HK, Finerman GA (1989) Autogeneic cancellous bone grafts in extensive segmental ulnar defects in dogs. Effects of xenogeneic bovine bone morphogenetic protein without and with interposition of soft tissues and interruption of blood supply. Clin Orthop Relat Res: 254–265. 2656028

[42] Sculean A, Blaes A, Arweiler N, Reich E, Donos N, Brecx M (2001) The effect of postsurgical antibiotics on the healing of intrabony defects following treatment with enamel matrix proteins. J Periodontol 72: 190–195. 11288792 10.1902/jop.2001.72.2.190

[43] Kaito T, Myoui A, Takaoka K, Saito N, Nishikawa M, Tamai N, et al (2005) Potentiation of the activity of bone morphogenetic protein-2 in bone regeneration by a PLA-PEG/hydroxyapatite composite. Biomaterials 26: 73–79. 15193882 10.1016/j.biomaterials.2004.02.010

[44] Meinig RP (2002) Polylactide membranes in the treatment of segmental diaphyseal defects: animal model experiments in the rabbit radius, sheep tibia, Yucatan minipig radius, and goat tibia. Injury 33 Suppl 2: B58–B65. 10.1016/s0020-1383(02)00133-x 12365366

[45] Beck LS, Wong RL, DeGuzman L, Lee WP, Ongpipattanakul B, Nguyen TH (1998) Combination of bone marrow and TGF-beta1 augment the healing of critical-sized bone defects. J Pharm Sci 87: 1379–1386. 9811494 10.1021/js9800883

[46] Kaweblum M, Aguilar MC, Blancas E, Kaweblum J, Lehman WB, Grant AD, et al (1994) Histological and radiographic determination of the age of physeal closure of the distal femur, proximal tibia, and proximal fibula of the New Zealand white rabbit. J Orthop Res 12: 747–749. 7931793 10.1002/jor.1100120519

[47] Masoud I, Shapiro F, Kent R, Moses A (1986) A longitudinal study of the growth of the New Zealand white rabbit: cumulative and biweekly incremental growth rates for body length, body weight, femoral length, and tibial length. J Orthop Res 4: 221–231. 3712130 10.1002/jor.1100040211

[48] Rivas R, Shapiro F (2002) Structural stages in the development of the long bones and epiphyses: a study in the New Zealand white rabbit. J Bone Joint Surg Am 84-A: 85–100. 11792784 10.2106/00004623-200201000-00013

[49] Bosch C, Melsen B, Vargervik K (1998) Importance of the critical-size bone defect in testing bone-regenerating materials. J Craniofac Surg 9: 310–316. 9780924 10.1097/00001665-199807000-00004

[50] Fernandez-Tresguerres-Hernandez-Gil I, Alobera-Gracia MA, Del-Canto-Pingarron M, Blanco-Jerez L (2006) Physiological bases of bone regeneration II. The remodeling process. Med Oral Patol Oral Cir Bucal 11: E151–E157. 16505794

[51] Kokubu T, Hak DJ, Hazelwood SJ, Hari Reddi A (2003) Development of an atrophic nonunion model and comparison to a closed healing fracture in rat femur. Journal of Orthopaedic Research 21: 503–510. 12706024 10.1016/S0736-0266(02)00209-7

[52] Bostrom M, Lane JM, Tomin E, Browne M, Berberian W, Turek T, et al (1996) Use of bone morphogenetic protein-2 in the rabbit ulnar nonunion model. Clin Orthop Relat Res: 272–282. 8641074 10.1097/00003086-199606000-00034

[53] Iwaniec UT, Wronski TJ, Liu J, Rivera MF, Arzaga RR, Hansen G, et al (2007) PTH stimulates bone formation in mice deficient in Lrp5. J Bone Miner Res 22: 394–402. 17147489 10.1359/jbmr.061118

[54] Novotny SA, Mader TL, Greising AG, Lin AS, Guldberg RE, Warren GL, et al (2014) Low intensity, high frequency vibration training to improve musculoskeletal function in a mouse model of Duchenne muscular dystrophy. PLoS One 9: e104339 10.1371/journal.pone.0104339 25121503 PMC4133244

